# Evaluating the prognostic value of CD56 in pediatric acute myeloid leukemia

**DOI:** 10.1186/s12885-022-10460-3

**Published:** 2022-12-21

**Authors:** Tianqi Liang, Zhiyong Peng, Chunfu Li, Junbin Huang, Huabin Wang, Chaoke Bu, Jian Li, Yongzhi Zheng, Xiaoqin Feng, Huiping Li, Chun Chen

**Affiliations:** 1 Division of Hematology/Oncology, Department of Pediatrics, The Seventh Affiliated Hospital, Sun Yat-sen University, Shenzhen, 518107 China; 2 Nanfang-Chunfu Children’s Institute of Hematology, Taixin Hospital, Dongguan, China; 3 Department of Hematology, Fujian Institute of Hematology, Fujian Provincial Key Laboratory on Hematology, Fujian Medical University Union Hospital, Fuzhou, China; 4 Department of Pediatrics, Nanfang Hospital, Southern Medical University, Guangzhou, China

**Keywords:** Acute myeloid leukemia, CD56, Prognostic value, Risk stratification, Immunophenotype, Cytogenetic abnormality

## Abstract

**Background:**

Many cytogenetic changes and gene mutations are associated with acute myeloid leukemia (AML) survival outcomes. CD56 is related to poor prognosis when expressed in adult AML patients. However, the prognostic value of CD56 in children with AML has rarely been reported. In this research, we aimed to evaluate the prognostic value of CD56 in childhood AML.

**Methods:**

The present retrospective study included 145 newly diagnosed pediatric patients with de novo AML (excluding AML-M3) in two hospitals between January 2015 and April 2021.

**Results:**

The total median (range) age was 75 (8–176) months, and the median follow-up time was 35 months. No significant difference in the 3-year overall survival rate was noted between the CD56-positive and CD56-negative groups (67.0% vs. 79.3%, *P* = 0.157) who received chemotherapy. However, among high-risk patients, the CD56-positive group had a worse overall survival rate and event-free survival rate (*P* < 0.05). Furthermore, among high-risk patients, the CD56-positive group had higher relapse and mortality rates than the CD56-negative group (*P* < 0.05).

**Conclusions:**

CD56 represents a potential factor of poor prognosis in specific groups of children with AML and should be considered in the risk stratification of the disease. Given the independent prognostic value of CD56 expression, we should consider integrating this marker with some immunophenotypic or cytogenetic abnormalities for comprehensive analysis.

**Supplementary Information:**

The online version contains supplementary material available at 10.1186/s12885-022-10460-3.

## Background

Acute myeloid leukemia (AML) is a malignant tumor originating from hematopoietic stem cells and is characterized by clonal proliferation and abnormal differentiation of myeloid cells in bone marrow and peripheral blood [1]. The morbidity and mortality of AML are high in both adults and children, and the overall survival (OS) rate of patients is relatively low [2]. To obtain a better therapeutic effect, the treatment protocol should be tailored to the risk profile of AML patients. For patients with a standard risk stratification, intensification of chemotherapy, including induction and consolidation, is the main treatment for AML in children [3]. For those with poor prognosis, especially refractory or recurrent patients, hematopoietic stem cell transplantation (HSCT) may represent an effective treatment [4]. Therefore, scientific stratification of AML and identification of patients with poor prognosis and modification of the treatment regimen as soon as possible are urgently needed.

At present, in addition to the chemotherapy response, cytogenetic characteristics and molecular profiling have become increasingly important for predicting patient prognosis. These methods are used to stratify and guide the treatment of patients with AML in clinical practice [5, 6]. Further incorporation of genomic and molecular data in pediatric AML will be helpful for additional refinements of risk stratification to enable tailoring of the treatment intensity [7]. CD56 is an isoform of neural cell adhesion molecule (NCAM), which is a glycoprotein of the immunoglobulin (Ig) superfamily expressed on the surface of various cells [8]. CD56 is expressed in up to 20% of AML cases, promotes the survival of tumor cells and improves their drug resistance [9]. A meta-analysis suggested that CD56 overexpression may represent a factor predicting poor prognosis in adult AML [10]. However, it has not been reported whether CD56 can be used to predict the prognosis of children with AML. Therefore, in our study, we retrospectively analyzed the effect of CD56 on prognosis in a large cohort of pediatric patients with AML treated at two centers of the cooperation group to evaluate the prognostic value of CD56 in childhood AML.

## Methods

### Patients

All procedures performed in studies involving human participants were in accordance with the ethical standards of the institutional and/or national research committee and with the 1964 Helsinki. This study has been approved by the Ethic Committee of the Nanfang Hospital of Southern Medical University. Informed written consent was obtained from all of the children and/or parents before study inclusion. This retrospective cohort study enrolled 150 children newly diagnosed with AML (non-M3). Two patients were excluded due to loss to follow-up, and three patients did not complete induction chemotherapy. Ultimately, this research included 145 children treated in the Department of Pediatrics, Nanfang Hospital of Southern Medical University and Fujian Medical University Union Hospital, China from January 2015 to April 2021. The diagnostic criteria and AML subtype were based on the 2008 World Health Organization (WHO) AML criteria. Patients with a second tumor and myeloid blast crisis phase of chronic myeloid leukemia were excluded. Blood was extracted from bone marrow at the first diagnosis for the detection of CD56 expression and cytogenetic abnormalities. According to the characteristics of genetic abnormality and the response to induction chemotherapy, all patients were classified according to risk stratification based on the C-HUANAN-AML15 protocol (Supplement 1).

### Flow cytometry

Flow cytometry was performed using bone marrow samples taken at diagnosis and analyzed in the specialized laboratory. Cells were stained with anti-CD45 (mAb), gated by CD45 expression and analyzed by flow cytometry. Plots were created based on the CD45 fluorescence intensity and side scattered (SSC) light. Cells were additionally stained with fluorescein-conjugated mAb against CD2, CD3, CD4, CD10, CD13, CD15, CD22, CD33, CD34, CD56, CD64, CD117, CD123, CD11b, CD19, CD20 and HLA-DR surface antigens. Antigen-negative subpopulations of cells were used as negative controls. The blast population was gated using scatter parameters, and antigen expression was rated positive when greater than 20% of AML cells expressed a specific antigen [11].

### Cytogenetic abnormality screening

Real-time polymerase chain reaction (RT–PCR) technology and G-banding technology were used for cytogenetic abnormality detection and chromosome karyotype analysis by Guangzhou KingMed Center for Clinical Laboratory.

### Chemotherapy treatment

All patients were treated according to the C-HUANAN-AML15 protocol. Based on the protocol, patients received induction and consolidation therapy. Induction therapy included two tandem courses of the FLAG-IDA regimen (course 1 and course 2): fludarabine (30 mg/m^2^/d IV on days 2 to 6), cytarabine (2 g/m^2^/d IV on days 2 to 6), idarubicin (8 mg/m^2^/d IV on days 4 to 6), and glycosylated G-CSF (5 μg/kg/d Ih on days 1 to 7). Consolidation therapy included the HAE and MidAC regimens. The HAE (course 3) regimen was administered as follows: homoharringtonine (HHT) (3 mg/m^2^/d IV on days 1 to 5), cytarabine (100 mg/m^2^ q12h IV on days 1 to 7), and etoposide (100 mg/m^2^/d, IV on days 1 to 5). The MidAC (course 4) regimen was administered as follows: mitoxantrone (10 mg/m^2^/d IV on days 1 to 5) and cytarabine (1 g/m^2^ q12h IV on days 1 to 3).

### Evaluation timing and definition

For patients treated with chemotherapy, bone marrow examination was performed on days 18 ~ 21 after every treatment to evaluate the effectiveness. The measured outcomes included death, relapse, CR, CR with incomplete recovery (CRi), event-free survival (EFS) and overall survival (OS). The definitions used for response criteria are based on those provided by Cheson et al. [12]. Specifically, relapse was defined as bone marrow blasts> 5% or occurrence of extramedullary disease. CR was defined as bone marrow blasts< 5%, absence of blasts with Auer rods, absence of extramedullary disease, ANC > 1.0 × 10^9^/L, PLT > 100 × 10^9^/L, and red cell transfusion independence.

CRi was defined as all CR criteria except for ANC > 1.0 × 10^9^/L and PLT > 100 × 10^9^/L. Resistance was noted when CR or CRi was not reached after 2 courses of induction therapy. The outcome measures were defined according to a review [13]: OS was calculated from the start of chemotherapy until death or last follow-up. EFS was calculated from the date of entry into the study until the date of induction treatment failure, relapse from CR or CRi or death from any cause. If the status of patients was not known at the last follow-up, they were censored on the date they were last examined.

### Statistical analysis

Statistical analyses were performed using SPSS software version 26.0. Groups were compared using Gray’s test. Statistical analyses were performed using Fisher’s exact test for categorical variables and the Mann–Whitney U test for continuous variables. Kaplan–Meier curves were used to describe changes in OS and EFS, and log-rank tests were used to compare the differences in the survival curves. Univariate and multivariate Cox regression analyses were used to correct for the effects of other confounding factors on survival. The hazard ratio was calculated for only the variables included in the Cox regression model, and the factors not included in the model had no corresponding hazard ratios. *P* < 0.05 was considered significant.

## Results

### Clinical and biological characteristics

**Fig. 1:**
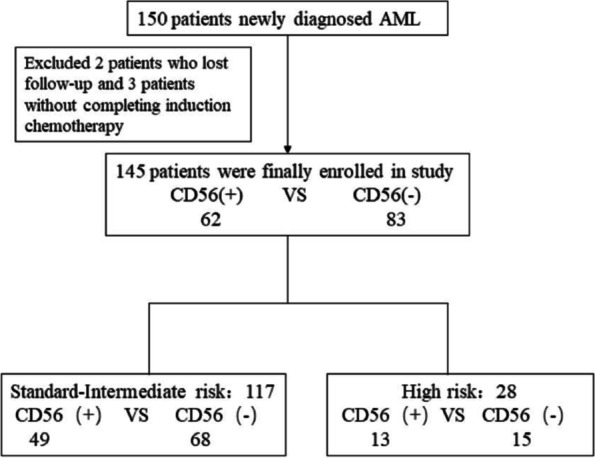
The study flowchart

A total of 145 patients (96.7%, 145/150) were enrolled in this retrospective study (Fig. 1). Among them, 62 were CD56 positive, and 83 were CD56 negative. The rate of CD56 positivity was approximately 42.8%. No differences in the distributions of age, sex, WBC count at diagnosis, extramedullary infiltration or risk stratification were noted (*P* > 0.05) (Table 1). Regarding biological features, there was no significant difference between the expression of CD56 and CD3, CD117, CD123 and CD34 in AML cells. In addition, no differences in the expression of AML1-ETO and FLT3-ITD mutations or chromosome karyotype abnormalities were noted between the two groups (*P* > 0.05) (Table 2).

**Table 1:** Clinical data and outcome of all patients according to CD56 expression

Patients data	CD56(+) (*n* = 62)	CD56(−) (*n* = 83)	*p*
Age(Months)			
Median(range)	64(10–176)	86(8–168)	0.110
≤ 3 years(n,%)	22(35.5%)	18(21.7%)	0.066
> 3 years(n,%)	40(64.5%)	65(78.3%)	
Sex(n,%)			
Male	31(50.0%)	52(62.7%)	0.128
Female	31(50.0%)	31(37.3%)	
WBC at diagnosis (n,%)			
< 50 × 10^9^/L	44(71.0%)	54(65.1%)	0.452
≥ 50 × 10^9^/L	18(29.0%)	29(34.9%)	
Extramedullary infiltration(n,%)			
Yes	6(9.7%)	7(8.4%)	0.795
No	56(90.3%)	76(91.6%)	
Risk stratification (n,%)			
Standard risk	8(12.9%)	18(21.7%)	0.391
Intermediate risk	41(66.1%)	50(60.2%)	
High risk	13(21.0%)	15(18.1%)	
FAB classification (n,%)			
M0	3(4.8%)	1(1.2%)	0.413
M1	2(3.2%)	0(0.0%)	
M2	19(30.6%)	23(27.7%)	
M4	2(3.2%)	3(3.6%)	
M5	24(38.7%)	43(51.8%)	
M6	1(1.6%)	2(2.4%)	
M7	3(4.8%)	3(3.6%)	
Unclassified	8(12.9%)	8(9.6%)	
Clinical outcome (n,%)			
CR1^a^	51(82.3%)	75(90.4%)	0.153
CR2^b^	54(87.1%)	80(96.4%)	0.076
Resistant	8(12.9%)	3(3.6%)	0.076
Relapse	7(11.3%)	5(6.0%)	0.255
Death	13(21.0%)	11(13.3%)	0.216

^a^CR after the first FLAG-IDA regimen

^b^CR after the two tandem courses of the FLAG-IDA regimen

**Table 2:** Biological features of patients according to CD56 expression

Biological features	CD56(+) No(%)	CD56(−) No(%)	*p*
AML1-ETO mutation(*n* = 145)			
Yes	21(33.9%)	24(28.9%)	0.523
No	41(66.1%)	59(71.1%)	
FLT3-ITD mutation(n = 145)			
Yes	4(6.5%)	9(10.8%)	0.360
No	58(93.5%)	74(89.2%)	
Complex karyotype(n = 145)			
Yes	9 (14.5%)	9 (10.8%)	0.507
No	53 (85.5%)	74 (89.2%)	
CD3(*n* = 119)			
Positive	21(39.6%)	27(40.9%)	0.887
Negative	32(60.4%)	39(59.1%)	
CD117(*n* = 123)			
Positive	49(89.1%)	64(94.1%)	0.495
Negative	6(10.9%)	4(5.9%)	
CD123(*n* = 97)			
Positive	38(95.0%)	50(87.7%)	0.389
Negative	2(5.0%)	7(12.3%)	
CD34(*n* = 121)			
Positive	41(77.4%)	58(85.3%)	0.261
Negative	12(22.6%)	10(14.7%)	

### Overall treatment results

**Fig. 2:**
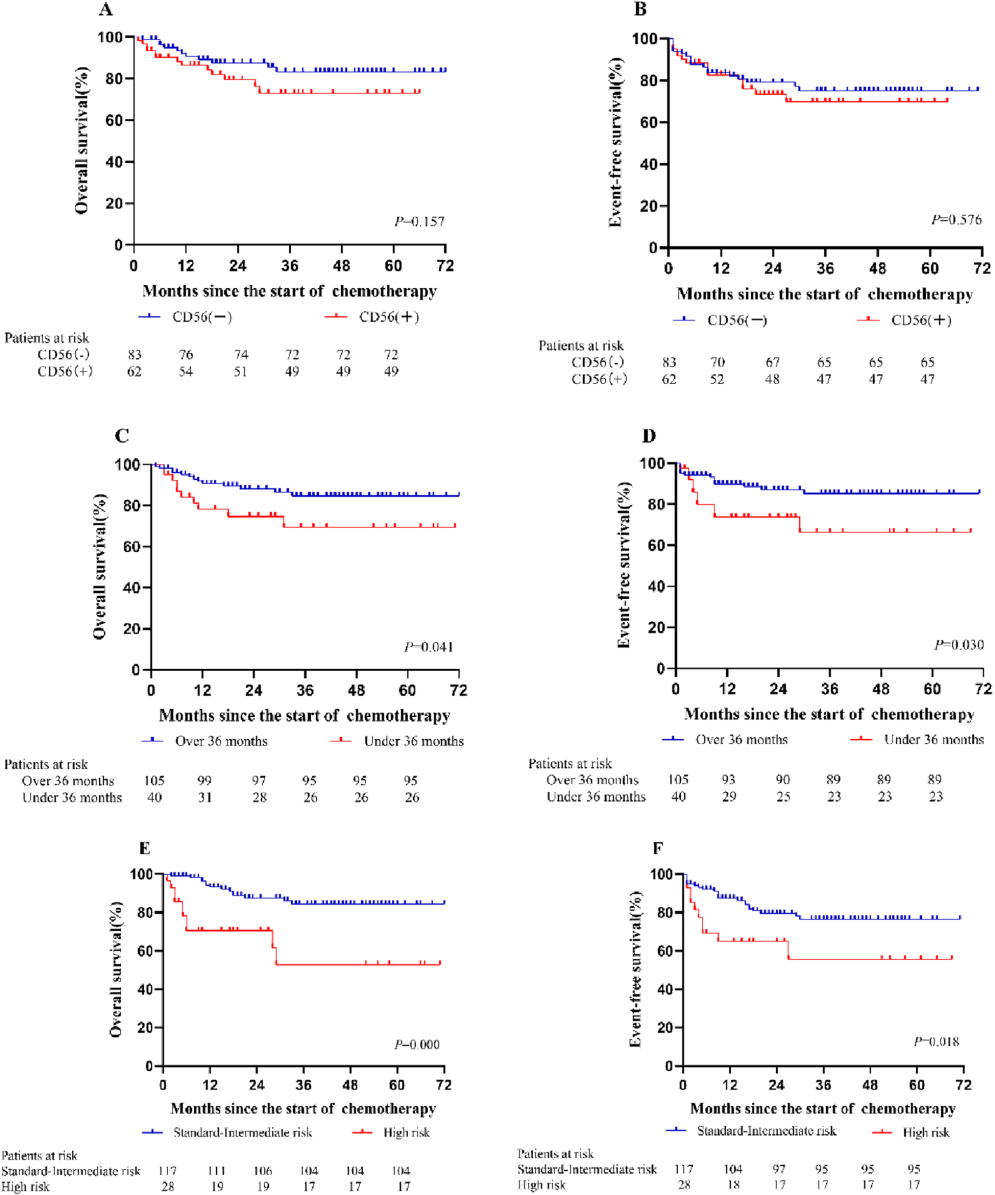
Kaplan–Meier analysis for risk factors of all enrolled patients. **A** The overall survival (OS) rate for patients in CD56 positive and CD56 negative group. **B** Event-free survival (EFS) rate for patients in CD56 positive and CD56 negative group. **C**: The OS rate in the group over 36 months. **D** The EFS rate in the group under 36 months. **E** The OS rate for patients in standard-intermediate risk group. **F** The EFS rate for patients in high risk group

According to clinical outcomes, no differences in the remission rates after the first induction chemotherapy and the second induction chemotherapy were noted between the CD56-positive group and the CD56-negative group. In addition, no significant differences in the overall resistance rate, relapse rate or mortality were noted between the two groups (*P* > 0.05) (Table 1). The median follow-up time was 35 months. According to the Kaplan–Meier analysis, in the CD56-positive group, the 3-year OS and EFS were 67 and 62.4%, respectively. In the CD56-negative group, the 3-year OS and EFS were 79.3 and 65%, respectively. No significant differences in OS or EFS were noted between the two groups (*P* > 0.05). Moreover, significantly lower OS and EFS were noted in patients less than 36 months old and in the high-risk group (*P* < 0.05) (Fig. 2).

### Univariate and multivariate analysis

According to the univariate analysis of risk factors, age ≤ 36 months at first diagnosis (HR 4.40, 95% CI: 1.95–9.93, *P* = 0.000), high-risk classification (HR 4.46, 95% CI: 1.97–10.09, *P* = 0.000) and complex karyotype (HR 3.34, 95% CI: 1.32–8.44, *P* = 0.011) were significantly associated with poor outcome. Regarding prognostic factors for EFS, age ≤ 36 months at first diagnosis (HR 3.42, 95% CI: 1.72–6.79, *P* = 0.000) and high-risk classification (HR 2.50, 95% CI: 1.19–5.28, *P* = 0.016) were independent risk factors. The AML1-ETO mutation may be a protective factor (HR 0.25, 95% CI: 0.09–0.72, *P* = 0.010). Multivariate analysis showed that only age ≤ 36 months at first diagnosis and high-risk classification were independent risk factors for OS and EFS (*P* < 0.05). However, in univariate and multivariate analyses, CD56 positivity was not related to OS or EFS (*P* > 0.05) (Table 3).

**Table 3:** Univariate and multivariate analyses of risk factors for overall survival and event-free survival in all enrolled patients

	OS of all patients		EFS of all patients	
	Univariate analysis			
Risk factors	HR (95% CI)	*P*	HR (95% CI)	*P*
≤36 months	4.40 (1.95–9.93)	0.000	3.42 (1.72–6.79)	0.000
High-risk	4.46(1.97–10.09)	0.000	2.50 (1.19–5.28)	0.016
CD56 positivity	1.77 (0.79–3.96)	0.164	1.21(0.61–2.41)	0.581
WBC ≥ 50 × 10^9^/L	1.17(0.50–2.74)	0.716	1.13(0.55–2.34)	0.736
AML1-ETO	0.38(0.13–1.10)	0.074	0.25(0.09–0.72)	0.010
FLT3-ITD	0.04(0.00–32.01)	0.353	1.33(0.41–4.37)	0.636
Complex karyotype	3.34(1.32–8.44)	0.011	2.04(0.84–4.95)	0.177
	Multivariate analysis			
Risk factors	HR (95% CI)	*P*	HR (95% CI)	*P*
≤36 months	2.85(1.08–7.50)	0.034	2.97(1.13–7.82)	0.028
High-risk	2.98(1.25–7.10)	0.013	2.74(1.15–6.56)	0.023
AML1-ETO	0.70(0.21–2.37)	0.570	0.56(0.21–2.35)	0.562
Complex karyotype	1.54(0.54–4.42)	0.420	1.25(0.47–3.33)	0.660

*Abbreviations*: *OS* overall survival, *EFS* event-free survival, *HR* hazard ratio, *CI* confidence interval, *WBC* white blood cell count at first diagnosis

### CD56 expression and overall treatment results in the high-risk group

**Fig. 3:**
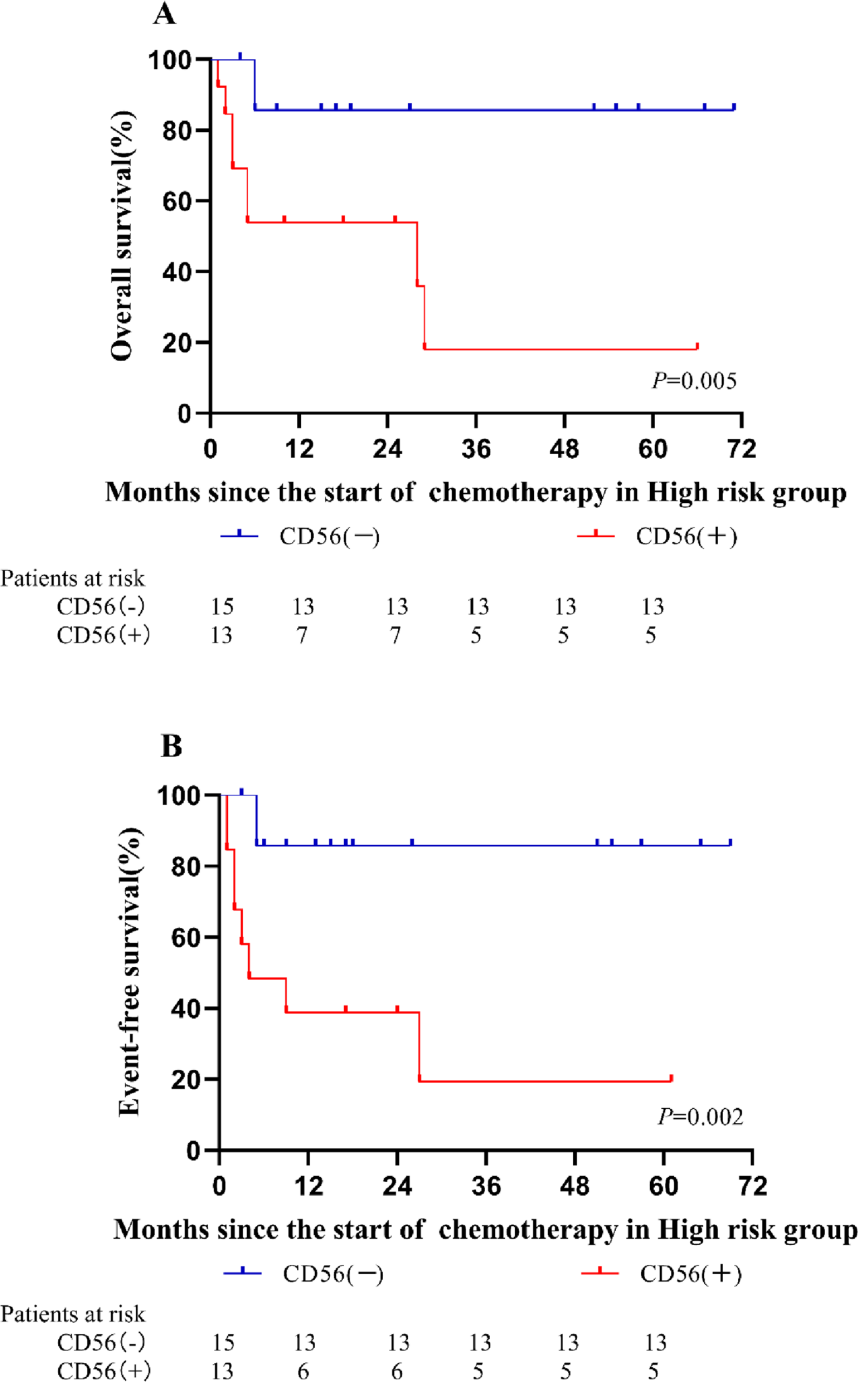
Kaplan–Meier analysis for the risk stratification for patients in High risk group. **A** The OS rate for patients in High risk group. **B** The EFS rate for patients in High risk group

There were a total of 28 patients in the high-risk group. Of these patients, 13 exhibited CD56 expression, whereas 15 did not. The rate of CD56 positivity was approximately 46.4%. This rate was slightly higher than the rate of CD56 expression in all enrolled patients (46.4% vs. 42.8%). No differences in the clinical data of all patients in the high-risk group were noted (*P* > 0.05). In the high-risk group, the response to chemotherapy was poor. After two courses of induction chemotherapy, only 5 patients achieved CR, and 8 patients developed resistance. The overall resistance rate and relapse rate were higher in the high-risk group compared with the standard-intermediate risk group (*P* = 0.05). By the end of the follow-up, 4 patients with CD56 expression relapsed, 8 of 13 patients died, and none of the patients without CD56 expression relapsed. The relapse rate and mortality of patients with CD56 expression were higher than their counterparts (*P* < 0.05) (Table 4). Furthermore, patients with CD56 expression had a worse OS rate and EFS rate (*P* < 0.05) (Fig. 3).

**Table 4:** Clinical data of Standard-Intermediate and High risk groups according to CD56 expression

Patients data	Standard-Intermediate risk			High risk		
	CD56(+) (*n* = 49)	CD56(−) (*n* = 68)	*p*	CD56(+) (*n* = 13)	CD56(−) (*n* = 15)	*P*
Age(Months)						
Median(range)	67(11–176)	87(8–168)	0.247	22(10–141)	38(8–162)	0.274
≤ 36 months(n,%)	14(28.6%)	11(16.2%)	0.107	8(61.5%)	8(53.3%)	0.476
> 36 months(n,%)	35(71.4%)	57(83.8%)		5(38.5%)	7(46.7%)	
Sex(n,%)						
Male	24(49.0%)	23(33.8%)	0.099	6(46.2%)	7 (46.7%)	1.000
Female	25(51.0%)	45(66.2%)		7(53.8%)	8 (53.3%)	
WBC at diagnosis (n,%)						
< 50 × 10^9^/L	32(65.3%)	44(64.7%)	0.946	12(92.3%)	10(66.7%)	0.173
≥ 50 × 10^9^/L	17(34.7%)	24(35.3%)		1(7.7%)	5(33.3%)	
Extramedullary infiltration(n,%)						
Yes	6(12.2%)	7(10.3%)	0.740	0(0.0%)	0(0.0%)	–
No	43(87.8%)	61(89.7%)		13(100%)	15(100.0%)	
AML1-ETO(n,%)						
Yes(n)	17(34.7%)	21(30.9%)	0.644	4(30.8%)	3(20.0%)	0.670
No(n)	32(65.3%)	47(69.1%)		9(69.2.9%)	12(80.0%)	
FLT3-ITD (n,%)						
Yes	3(6.1%)	6(8.8%)	0.850	1(7.7%)	3(20.0%)	0.600
No	46(93.9%)	62(91.2%)		12(92.3%)	12(80.0%)	
Complex karyotype(n,%)						
Yes	4(8.2%)	1(1.5%)	0.160	5 (38.5%)	8 (53.3%)	0.476
No	45(91.8%)	67(98.5%)		8 (61.5%)	7 (46.7%)	
Clinical outcome (n,%)						
CR1^a^	48(98.0%)	67(98.5%)	1.000	3(23.1%)	8 (53.3%)	0.137
CR2^b^	49(100.0%)	68(100.0%)	–	5(38.5%)	12 (80.0%)	0.050
Resistant	0	0	–	8 (61.5%)	3 (20.0%)	0.050
Relapse	3(6.1%)	5(7.4%)	1.000	4(30.8%)	0 (0.0%)	0.035
Death	5(10.2%)	9(13.2%)	0.618	8(61.5%)	2 (13.3%)	0.016

^a^CR after the first FLAG-IDA regimen

^b^CR after the two tandem courses of the FLAG-IDA regimen

## Discussion

AML is one of the most challenging diseases in children with malignant tumors. Although advances in therapy have been made over the past decades, the OS of children with AML has not been satisfactory [7]. Despite the availability of different treatments for childhood AML, the therapeutic effects vary greatly [14, 15]. To improve the curative effect, the response to chemotherapy and risk stratification should be seriously considered. When considering risk stratification, it is necessary to combine cytogenetic characteristics and molecular disease features. A previous study reported the relationship between outcome and the expression of a single antigen [16]. The abnormal immunophenotype on the surface of AML cells affects survival outcome. Therefore, it is very important to identify a new antigen marker that is highly expressed on the surface of AML cells and related to the clinical outcome.

CD56, which is also known as NCAM1, is abnormally expressed in 15 to 20% of patients with AML and is associated with reduced complete remission rates, high relapse rates and poor OS [17]. Laboratory research has shown that CD56 expression promotes leukemogenesis and confers drug resistance in AML [9]. In adults, clinical studies have found that CD56 indicates poor prognosis in different AML subtypes [18–20]. Therefore, CD56 overexpression is an adverse prognostic factor for AML in adults. However, large clinical studies confirming the role of CD56 in childhood AML are lacking. The purpose of our research was to verify whether this association exists.

Yusuke Hara et al. reported that the prognosis of children with AML was worse in the group of children less than 3 years old [21]. In addition, we also found that OS and EFS were worse in children less than 36 months old, which was an independent risk factor for poor prognosis. In this group, the proportion of CD56-positive patients tended to increase, indicating that CD56 expression in children with AML may be related to age. The lack of a significant difference may be due to the small sample size of our study. Concerning the biologic features of CD56-positive patients, CD3, CD117 and CD34, which belong to hematopoietic stem and progenitor cells and T-cell antigens were not coexpressed with CD56 in AML patients [22]. This finding suggested that CD56-positive AML cells may not appear in progenitor cells that are not restricted by lineage. In addition, CD56 was not coexpressed with the immaturity-associated marker CD123 in common hematological tumors [23], suggesting that CD56 may not be used as an immune-related marker for the diagnosis of AML.

CD56-positive patients had a lower induced remission rate and higher relapse and mortality rates in the high-risk group with childhood AML. However, CD56 expression did not affect overall OS or EFS. Based on the risk stratification of our study, the high-risk group was a group of patients with a poor response to chemotherapy or specific cytogenetic abnormalities. This finding indicated that the poor prognosis associated with CD56 expression may only occur in a specific population. Laura *M. Pardo* et al. found that in CD56-positive AML, poor prognosis is limited to a subset of patients with unique multidimensional phenotypes [24]. The above results suggested that CD56 may affect the survival outcome in specific AML groups or when some multidimensional immunophenotypic and cytogenetic abnormalities are present. Unfortunately, our study did not identify other related immaturity-associated markers or cytogenetic abnormalities.

There are some limitations in the study. First, specific antigens and gene mutations were not assessed in all patients upon enrollment, resulting in incomplete immunophenotype and gene mutation data in some patients. Second, as a retrospective study, the number of enrolled patients in this study was still not sufficient, and the follow-up time needs to be further extended.

In conclusion, our research showed that CD56 can be used as one of the factors of poor prognosis in specific groups of children with AML, which should be considered in the risk stratification of the disease. In addition to focusing on the independent prognostic value of CD56 expression, we should consider integrating this marker into a multidimensional immunophenotype or cytogenetic abnormalities for comprehensive analysis, which is more helpful to scientifically classify and evaluate the prognosis of children with AML.

## Supplementary Information

**Additional file 1:** **Supplement 1.** Risk stratification based on the C-HUANAN-AML15 protocol.

## Data Availability

The datasets used and/or analysed during the current study available from the corresponding author on reasonable request.

## Abbreviations

OS: overall survival
EFS: event-free survival
HR: hazard ratio
CI: confidence interval
WBC: white blood cell count
ANC: absolute neutrophil count
PLT: platelet count
CR: complete recovery
CRi: CR with incomplete recovery
AML: acute myeloid leukemia
AML-M3: acute promyelocytic leukemia
HSCT: hematopoietic stem cell transplantation
NCAM: neural cell adhesion molecule
RT-PCR: real-time polymerase chain reaction
mAb: monoclonal antibody

## References

[1] Short NJ, Rytting ME, Cortes JE (2018). Acute myeloid leukaemia. Lancet.

[2] De Kouchkovsky I, Abdul-Hay M (2016). Acute myeloid leukemia: a comprehensive review and 2016 update. Blood Cancer J.

[3] Zwaan CM, Kolb EA, Reinhardt D, Abrahamsson J, Adachi S, Aplenc R, De Bont ES, De Moerloose B, Dworzak M, Gibson BE (2015). Collaborative efforts driving Progress in pediatric acute myeloid leukemia. J Clin Oncol.

[4] Zuckerman T, Rowe JM (2014). Transplantation in acute myeloid leukemia. Hematol Oncol Clin North Am.

[5] Bochtler T, Stolzel F, Heilig CE, Kunz C, Mohr B, Jauch A, Janssen JW, Kramer M, Benner A, Bornhauser M (2013). Clonal heterogeneity as detected by metaphase karyotyping is an indicator of poor prognosis in acute myeloid leukemia. J Clin Oncol.

[6] Dohner H, Estey E, Grimwade D, Amadori S, Appelbaum FR, Buchner T, Dombret H, Ebert BL, Fenaux P, Larson RA (2017). Diagnosis and management of AML in adults: 2017 ELN recommendations from an international expert panel. Blood.

[7] Elgarten CW, Aplenc R (2020). Pediatric acute myeloid leukemia: updates on biology, risk stratification, and therapy. Curr Opin Pediatr.

[8] Valgardsdottir R, Capitanio C, Texido G, Pende D, Cantoni C, Pesenti E, Rambaldi A, Golay J, Introna M (2014). Direct involvement of CD56 in cytokine-induced killer-mediated lysis of CD56+ hematopoietic target cells. Exp Hematol.

[9] Sasca D, Szybinski J, Schuler A, Shah V, Heidelberger J, Haehnel PS, Dolnik A, Kriege O, Fehr EM, Gebhardt WH (2019). NCAM1 (CD56) promotes leukemogenesis and confers drug resistance in AML. Blood.

[10] Xu S, Li X, Zhang J, Chen J (2015). Prognostic value of CD56 in patients with acute myeloid leukemia: a meta-analysis. J Cancer Res Clin Oncol.

[11] Bene MC, Castoldi G, Knapp W (1995). Proposals for the immunological classification of acute leukemias. European Group for the Immunological Characterization of Leukemias (EGIL). Leukemia.

[12] Creutzig U, Kaspers GJ (2004). Revised recommendations of the international working group for diagnosis, standardization of response criteria, treatment outcomes, and reporting standards for therapeutic trials in acute myeloid leukemia. J Clin Oncol.

[13] Dohner H, Estey EH, Amadori S, Appelbaum FR, Buchner T, Burnett AK, Dombret H, Fenaux P, Grimwade D, Larson RA (2010). Diagnosis and management of acute myeloid leukemia in adults: recommendations from an international expert panel, on behalf of the European LeukemiaNet. Blood.

[14] Rasche M, Zimmermann M, Borschel L, Bourquin JP, Dworzak M, Klingebiel T, Lehrnbecher T, Creutzig U, Klusmann JH, Reinhardt D (2018). Successes and challenges in the treatment of pediatric acute myeloid leukemia: a retrospective analysis of the AML-BFM trials from 1987 to 2012. Leukemia.

[15] Pession A, Masetti R, Rizzari C, Putti MC, Casale F, Fagioli F, Luciani M, Lo Nigro L, Menna G, Micalizzi C (2013). Results of the AIEOP AML 2002/01 multicenter prospective trial for the treatment of children with acute myeloid leukemia. Blood.

[16] Casasnovas RO, Slimane FK, Garand R, Faure GC, Campos L, Deneys V, Bernier M, Falkenrodt A, Lecalvez G, Maynadie M (2003). Immunological classification of acute myeloblastic leukemias: relevance to patient outcome. Leukemia.

[17] Sun Y, Wan J, Song Q, Luo C, Li X, Luo Y, Huang X, Ding R, Li H, Hou Y (2021). Prognostic significance of CD56 antigen expression in patients with De novo non-M3 acute myeloid leukemia. Biomed Res Int.

[18] Iriyama N, Hatta Y, Takeuchi J, Ogawa Y, Ohtake S, Sakura T, Mitani K, Ishida F, Takahashi M, Maeda T (2013). CD56 expression is an independent prognostic factor for relapse in acute myeloid leukemia with t(8;21). Leuk Res.

[19] Ono T, Takeshita A, Kishimoto Y, Kiyoi H, Okada M, Yamauchi T, Emi N, Horikawa K, Matsuda M, Shinagawa K (2014). Expression of CD56 is an unfavorable prognostic factor for acute promyelocytic leukemia with higher initial white blood cell counts. Cancer Sci.

[20] Montesinos P, Rayon C, Vellenga E, Brunet S, Gonzalez J, Gonzalez M, Holowiecka A, Esteve J, Bergua J, Gonzalez JD (2011). Clinical significance of CD56 expression in patients with acute promyelocytic leukemia treated with all-trans retinoic acid and anthracycline-based regimens. Blood.

[21] Hara Y, Shiba N, Yamato G, Ohki K, Tabuchi K, Sotomatsu M, Tomizawa D, Kinoshita A, Arakawa H, Saito AM (2020). Patients aged less than 3 years with acute myeloid leukaemia characterize a molecularly and clinically distinct subgroup. Br J Haematol.

[22] Lennartsson J, Ronnstrand L (2012). Stem cell factor receptor/c-kit: from basic science to clinical implications. Physiol Rev.

[23] Testa U, Pelosi E, Castelli G. CD123 as a therapeutic target in the treatment of hematological malignancies. Cancers. 2019;11(9). 10.3390/cancers11091358 PMC6769702 31547472

[24] Pardo LM, Voigt AP, Alonzo TA, Wilson ER, Gerbing RB, Paine DJ, Dai F, Menssen AJ, Raimondi SC, Hirsch BA (2020). Deciphering the significance of CD56 expression in pediatric acute myeloid leukemia: a report from the Children's oncology group. Cytometry B Clin Cytom.

