# Supplementary material for: Evaluating the prognostic value of CD56 in pediatric acute myeloid leukemia
Source: BMC Cancer. 2022 Dec 21;22:1339. doi: 10.1186/s12885-022-10460-3 (PMC9768963; doi:10.1186/s12885-022-10460-3)
Supplement: Supplementary file 1 — Additional file 1: Supplement 1. Risk stratification based on the C-HUANAN-AML15 protocol. [file 12885_2022_10460_MOESM1_ESM.doc]

Supplement 1. Risk stratification based on the C-HUANAN-AML15 protocol

| Based on genetic abnormality and induction chemotherapy response | |
| --- | --- |
| Standard-risk criteria | Including one of the following genetic abnormalities and CR after Course 1:  1. t(8:21)(q22:q22); AML/ETO (RUNX1-RUNX1T1)  2. inv(16)(p13q22)/t(16:16)(p13:q22); CBFβ-MYH11(+)  3. Normal karyotype; NPM1 positivity or double CEBPA mutation in the absence of FLT3-ITD |
| Intermediate-risk criteria | No genetic abnormality according to standard and high risk criteria and blasts in bone marrow <15% after Course 1 and CR after Course 2 |
| High-risk criteria | Including one of the following genetic abnormalities or/and blasts in bone marrow ≥15% after Course 1 or no CR after Course 2:  1. Mutated FLT3-ITD  2. Complex karyotype  3. -5 or del5(q)  4. abn(3q)  5. abn(17p)  6. -7 or del(7q) |
